# Novel Insights into the Role of Long Noncoding RNA in Ocular Diseases

**DOI:** 10.3390/ijms17040478

**Published:** 2016-03-31

**Authors:** Fang Li, Xuyang Wen, He Zhang, Xianqun Fan

**Affiliations:** Department of Ophthalmology, Ninth People’s Hospital, School of Medicine, Shanghai JiaoTong University, Shanghai 200025, China; lifangniuniu@126.com (F.L.); winston-a@hotmail.com (X.W.)

**Keywords:** epigenetics, long noncoding RNA, ocular disease

## Abstract

Recent advances have suggested that long noncoding RNAs (lncRNAs) are differentially expressed in ocular tissues and play a critical role in the pathogenesis of different types of eye diseases. Here, we summarize the functions and mechanisms of known aberrantly-expressed lncRNAs and present a brief overview of relevant reports about lncRNAs in such ocular diseases as glaucoma, proliferative vitreoretinopathy (PVR), diabeticretinopathy (DR), and ocular tumors. We intend to highlight comprehensive studies that provide detailed data about the mechanisms of lncRNAs, their applications as diagnostic or prognostic biomarkers, and their potential therapeutic targets. Although our understanding of lncRNAs is still in its infancy, these examples may provide helpful insights into the methods by which lncRNAs interfere with ocular diseases.

## 1. Introduction

The dramatically increasing prevalence of ocular disorders worldwide, including ocular diseases that lead to visual impairment and eventual blindness, such as glaucoma, retinal degeneration, and ocular tumors, will likely continue, particularly in underdeveloped countries but also in developed regions. Ocular diseases can seriously affect an individual’s health and quality of life, while also imposing a substantial emotional, medical, and economic burden on patients and society [1]. Thus, early detection and prompt treatment is of great importance to prevent visual impairment by disease. To date, the occurrence and development of eye diseases have been primarily attributed to specific gene mutations, such as *RB1* for retinoblastoma [2,3] and *GNAQ*, *GNA11*, *EIF1AX*, *SF3B1*, *BAP1*, and *PLCB4* for uveal melanoma [4,5,6,7]. However, some cases include non-Mendelian distributions, and their variability remains unaccounted for by conventional risk factors or genetics. Epigenetics has recently emerged as an increasingly powerful paradigm for understanding and potentially explaining the onset and progression of some ocular diseases. In addition, epigenetic alterations are generally reversible, which will undoubtedly make them attractive targets for potential new epigenetic therapies in the future. Epigenetic modifications mainly include DNA methylation, histone modifications, chromatin structure, and noncoding RNAs. In this review, we focus on the most recently discovered class, namely long noncoding RNAs (lncRNAs).

LncRNAs are recognized as transcripts that are longer than 200 nucleotides and that structurally resemble mRNA but have little or no protein-coding potential. Most lncRNAs are located in the nucleus, but a substantial minority of nearly 15% are located in the cytoplasm [8]. According to their genomic locations, lncRNAs can be divided into several types [9]. Certain lncRNAs that overlap with, or are antisense transcribed to, protein-coding genes are defined as sense or antisense. A lncRNA can also be bidirectional if its promoter and a coding transcript are in close proximity, oriented in a head-to-head fashion. Intronic lncRNAs are genes derived from the introns of protein-coding genes, and the term lincRNA refers to a lncRNA located within an intergenic region of the genome [10,11,12]. Compared with linear ncRNA, circular RNA (circRNA) represents a distinct group of RNA molecules that are longer than 200 nucleotides [13].

Studies have shown that lncRNAs play important regulatory roles in multiple biological processes, such as stem cell maintenance, cell lineage commitment, and cellular phenotype differentiation [14,15,16]. Most lncRNAs exert a broad influence on transcriptional regulation through several modes, including signal, decoy, guide, and scaffold [17]. Briefly, a lncRNA may act as a signal in response to various stimuli, recruiting corresponding complexes to activate or silence gene expression. Additionally, a lncRNA may guide or sequester transcription factors to bind to a specific site of action, or it may interact with multiple components, thereby repressing or activating gene expression [18,19,20]. Additionally, some lncRNAs may affect gene expression through post-transcriptional events [21]. LncRNAs can enhance or reduce protein translation via mRNA alternative splicing, turnover, export, and translocation, or they may reduce the effect of microRNAs (miRNAs) on mRNA stability by acting as competing endogenous RNAs or RNA sponges when they contain an miRNA-binding sequence [22,23]. In addition, lncRNAs also participate in the post-translational modification of proteins.

Defining the functions and potential mechanisms of lncRNAs has been the focus of recent and intense research. To date, several lncRNAs have been implicated in common ocular diseases, such as corneal vascularization, glaucoma, proliferative vitreoretinopathy, diabetic retinopathy, and ocular tumors, among others. However, the functions and detailed mechanisms by which lncRNAs affect these diseases remain largely unknown. Here, we review and summarize the currently identified lncRNAs as follows (Table 1).

## 2. Roles of lncRNAs in Ocular Disease

### 2.1. Role of lncRNA in Corneal Neovascularization (CN)

Chronic hypoxia or various inflammatory stimuli, such as bacterial keratitis, alkaline burns, and graft rejections, can lead to corneal neovascularization, which results in visual impairment or even blindness [39]. Huang *et al.* [24] identified 154 differentially-expressed lncRNAs between vascularized and normal corneas, including 60 down-regulated lncRNAs and 94 up-regulated lncRNAs. The lncRNA *NR_033585* was significantly up-regulated in vascularized corneas and presented a similar expression pattern as pro-angiogenic factors, such as VEGF, MMP-9, and Ang-2, whereas the lincRNA *chr8:129102060–129109035* reverse strand was markedly down-regulated in vascularized corneas and showed a similar expression pattern to the anti-angiogenesis factor PDGF [40]. This study provides a novel insight into CN pathogenesis, namely that lncRNAs can perform pro-angiogenic or anti-angiogenicroles in vascularization, and dysregulated lncRNAs may, thus, become potential targets for prevention or treatment.

### 2.2. Role of lncRNAs in Glaucoma

Primary open-angle glaucoma (POAG) is the most frequent subtype of glaucoma, and it is characterized pathologically by a progressive loss of retinal ganglion cells and a corresponding loss of the visual field. Evidence from several studies has shown that genetic variants at the chromosome 9p21 locus, including *CDKN2B-AS1*, *CDKN2A*, and *CDKN2B* genes, are associated with POAG [25,26,41,42,43,44].

*CDKN2B-AS*, also known as *ANRIL*, is a lncRNA transcribed in the antisense direction of *CDKN2A* and *CDKN2B*. *ANRIL* is a well-established tumor suppressor whose function is disabled in human cancers [45]. Additionally, *ANRIL* has been widely implicated in increased susceptibility to many diseases, including coronary artery disease, myocardial infarction, type 2 diabetes, and Alzheimer disease [46,47]. The extensive roles of *ANRIL* in disease were discovered in a series of linkage studies, in which single-nucleotide polymorphisms (SNPs) in a region spanning 120 kb around the *INK4b-ARF-INK4a* locus were associated with disease. The molecular mechanisms underlying the association between *ANRIL* and POAG are not well understood [48]. One possible explanation is that the occurrence of polymorphisms at these loci alters the expression of target genes that regulate the cell cycle or acts through epigenetic mechanisms, subsequently inducing a tendency toward retinal ganglion cell apoptosis and glaucoma [25,26,27]. Another study identified associations between 9p21 variants and glaucoma features, suggesting that the *ANRIL* region modifies the vulnerability of the optic nerve to glaucomatous change, further implying a role of *ANRIL* in modulating optic nerve degeneration [42]. Compared with patients who lack glaucoma risk alleles, patients carrying the risk alleles have a lower intraocular pressure (IOP) and a larger vertical cup-to-disc ratio (VCDR) [49] and are predisposed to the development of POAG at lower IOP levels; in other words, these patients exhibit stronger associations with normal tension glaucoma (NTG) and advanced glaucoma phenotypes [44].

All of this evidence supports a key regulatory role for *ANRIL* in the development of glaucoma. POAG can be difficult to diagnose at early stages, and defining high- or low-risk alleles may be useful for the early determination of whether patients with suspected glaucoma should receive prioritized treatment to slow disease progression and avoid blindness.

### 2.3. Role of lncRNAs in Proliferative Vitreoretinopathy

Proliferative vitreoretinopathy (PVR) is a serious complication of retinal detachment and vitreoretinal surgery, and epiretinal membrane (ERM) formation leads to severe reductions in vision. Zhou *et al.* [28] performed a microarray to identify PVR-related lncRNAs, and the lncRNA *MALAT1* was found to be significantly up-regulated in the fibrovascular membrane. *MALAT1*, also known as nuclear-enriched transcript 2 (*NEAT2*), is a lncRNA that is highly expressed in individuals who are high risk for the metastasis of non-small cell lung tumors [50]. The increased expression of *MALAT1* has been associated with retinal pigment epithelium proliferation and migration, promotion of ERM formation, and PVR pathogenesis. Moreover, *MALAT1* is also up-regulated in peripheral blood samples from PVR patients, implying that it may represent an easily detectable biomarker for noninvasive diagnosis to identify high-risk PVR patients [28].

### 2.4. Role of lncRNAs in Diabeticretinopathy

Diabeticretinopathy (DR) is one of the most common vascular complications in patients with long-term diabetes. Visual deterioration is tightly related to retinal inflammation, retinal neovascularization, vascular hyperpermeability, and vascular cell apoptosis [51,52]. During the pathogenesis and progression of retinopathy, certain lncRNAs show a potential association.

Myocardial infarction-associated transcript (*MIAT*), also known as Gomafu or retinal noncoding RNA 2 (*RNCR2*), was first identified as a susceptibility locus for myocardial infarction patients [53] and is reportedly highly expressed in retinal precursor cells [54]. Yan *et al.* [29] showed that the *MIAT* level is clearly up-regulated following treatment with high-glucose or oxidative stress and this up-regulation contributes to endothelial cell proliferation and migration, thus leading to microvascular dysfunction. To further explore the therapeutic effects of *MIAT* in diabetic retinas, they also investigated the role of *MIAT* in cultured endothelial cells. Down-regulating *MIAT* using siRNA significantly inhibited endothelial inflammatory responses. The underlying mechanisms may be related to the role of *MIAT* as a competing endogenous RNA (ceRNA) in the regulation of VEGF levels, thereby promoting retinal neovascularization. The ceRNA phenomenon is a recently-proposed hypothesis in which all RNA transcripts that share miRNA-binding sites can communicate with, and regulate, each other by competing specifically for shared miRNAs [55]. *MIAT* can bind to the same site as miR-150-5p, thus alleviating the miR-150-5p repression effect and up-regulating the level of the miR-150-5p target gene *VEGF*. Moreover, *MIAT* knockdown inhibits the up-regulation of TNF-α and ICAM-1, thereby alleviating vascular leakage and inflammation; as these are the key features of different stages of DR, *MIAT* knockdown shows an impressive therapeutic benefit [56].

*MALAT1* is highly expressed in a wide range of tumors, including lung cancer, liver cancer, renal cell carcinoma, bladder cancer, and osteosarcoma, and it also participates in the pathogenesis of DR [57]. Yan *et al.* [30] performed lncRNA profiling in a murine model of DR using microarray analysis, and they identified 303 aberrantly expressed lncRNAs. *MALAT1* expression was significantly up-regulated in a RF/6A cell model of hyperglycemia, in aqueous humor samples, and in the fibrovascular membranes of diabetic patients. Moreover, experimental evidence has shown that *MALAT1* plays an important role in diabetes-induced retinal vessel dysfunction. Liu *et al.* [32] found that *MALAT1* regulated retinal endothelial cell function and pathological microvascular growth under diabetic conditions. Knockdown of *MALAT1* significantly alleviates diabetes-induced microvascular dysfunction *in vivo* and inhibits endothelial cell proliferation, migration, and tube formation *in vitro* by changing the levels of phosphorylated p38 MAPKs. Michalik *et al.* [31] also confirmed that genetic ablation of *MALAT1 in vivo* inhibits the proliferation of endothelial cells and reduces neonatal retinal vascularization.

All the above lines of evidence show that *MIAT* and *MALAT1* are involved in DR. Thus, both of these lncRNAs can help us understand the pathogenesis of DR, and they provide new, promising therapeutic targets for DR treatment in the future.

### 2.5. Role of lncRNAs in Choroidal Neovascularization

Choroidal neovascularization (CNV) is a hallmark of neovascular age-related macular degeneration (AMD), a leading cause of visual impairment in elderly individuals [58]. Xu *et al.* [33] found that the expression of VEGF and two lncRNAs, *Vax2os1* and *Vax2os2*, were significantly up-regulated in the aqueous humor of CNV patients, making them predictive biomarkers for the diagnosis of ocular neovascular diseases [59]. *Vax2os1* and *Vax2os2*, which are antisense transcripts of the *Vax2* gene, are highly expressed in the choroid and retinal vasculature. The strong RNA-protein interactions between *Vax2os1* and C1D and between *Vax2os2* and PATL2 play important roles in the mechanism underlying the pathogenesis of CNV because C1D and PATL2 are important for regulating the stability of chromatin structure [60,61,62]. Increased information about these two lncRNAs will facilitate a greater understanding of CNV pathogenesis. Provided that each lncRNA regulates specific facets of protein activity, a more refined and less toxic drug targeting a lncRNA may be employed for CNV treatment.

### 2.6. Roles of lncRNAs in Ocular Tumors

#### 2.6.1. Retinoblastoma

Retinoblastoma is a rare malignancy of the retina that usually appears before the age of five years, threatening the vision and survival of children if timely detection and treatment are not achieved. Therefore, to preserve vision, salvage the eye, and save the child’s life, elucidating the molecular mechanisms of retinoblastoma and identifying specific biomarkers for tumor progression are of utmost importance. However, only two lncRNAs, *BANCR* and *MEG3*, have been associated with retinoblastoma.

The involvement of BRAF-activated noncoding RNA (*BANCR*), a 693-bp lncRNA encoded on chromosome 9, in the proliferation and metastasis of malignant melanoma and lung cancer via the MAPK pathway has been reported [63,64,65]. *BANCR* has been shown to play a key role in gastric cancer cells via regulation of *NF-κB1* [66]. In retinoblastoma tissues and cell lines, recent evidence has shown that *BANCR* is over-expressed and is highly associated with tumor size, choroidal invasion, and optic nerve invasion. Knockdown of *BANCR* significantly suppresses the proliferation, migration, and invasion of retinoblastoma cells *in vitro*, thus implying a better prognosis [34].

Maternally-expressed gene 3 (*MEG3*), an imprinted gene located on chromosome 14q32 [67], is considered to act as a tumor suppressor lncRNA. The loss of *MEG3* expression in various human tumors has been well documented. Re-expression of *MEG3* inhibits proliferation, induces apoptosis, and inhibits the anchorage-independent growth of human tumor cells [68]. In retinoblastoma samples, Gao *et al.* [35] found that *MEG3* is significantly down-regulated and that the reduced expression is associated with a poor prognosis among retinoblastoma patients. Studies have shown that *MEG3* suppresses retinoblastoma progression by negatively regulating the Wnt/β-catenin pathway. Studies have also shown that pancreatic cancer cell proliferation could be inhibited via *MEG3*-mediated p53 activation [69], implying that *MEG3* is a potential molecular therapeutic target.

#### 2.6.2. Uveal Melanoma

Uveal melanoma is the most common eye malignancy in adults; it causes severe visual morbidity and is fatal to approximately 50% of patients. Fan *et al.* [36] found that the lncRNA *ROR* (retinoid-related orphan nuclear receptor) and its target gene *TESC* were both highly expressed relative to normal cells or adjacent normal tissues in three malignant ocular melanoma cell lines and in 20 ocular melanoma tissues. *ROR* acts as an oncogenic lncRNA, activating the *TESC* promoter by repelling the histone G9A methyltransferase and promoting the release of histone H3K9 methylation. Suppression of *ROR* could reduce tumor growth and metastasis.

*SF3B1* mutations are associated with a good prognosis for uveal melanoma. Recently, an RNA-seq analysis showed that mutations in *SF3B1* are associated with cryptic alternative splicing within exon 4 of *CRNDE*, indicating that this lncRNA has potential importance for determining how alternative splicing affects cellular function [37]. Evidence has shown that *CRNDE* can promote glioma cell growth and invasion through mTOR signaling, thereby highlighting the potential of *CRNDE* as a novel therapeutic target for the treatment of glioma [70]. In addition, several lines of evidence have shown that *CRNDE* exerts its effects on RNA transcripts primarily via epigenetic mechanisms, particularly through histone methylation or demethylation by the PRC2 or CoREST complexes, respectively [71]. The detailed mechanism of how lncRNA is involved in uveal melanoma remains to be studied; however, the results will undoubtedly contribute to knowledge of the uveal melanoma tumorigenesis and suggest new therapeutic strategies.

### 2.7. Roles of lncRNAs in Other Ocular Disease

MOMO syndrome, short for macrosomia, obesity, macrocephaly, and ocular abnormalities, is an extremely rare syndrome. The main features of ocular abnormalities include retinal coloboma, nystagmus, and downward-slanting palpebral fissures. Recently, one MOMO patient showed a homozygous, balanced, reciprocal translocation (16; 20) (q21; p11.23) that was inherited from healthy consanguineous parents. The breakpoint at 16q21 did not disrupt any known or predicted gene, whereas the chromosome 20 breakpoint disrupted a new lncRNA at 20p11.23 named *LINC00237*. Compared with control individuals, the expression of *LINC00237* was reduced by approximately 50% in patients’ lymphoblasts. This disruption causes gene inactivation that results in the loss of complete transcript production [38]. However, the function of this candidate gene and the consequences of its haploinsufficiency remain to be characterized.

The maintenance of corneal-specific epithelial qualities plays an important role in maintaining corneal transparency and preventing vision loss. PNN is a nuclear protein that is associated with the splicing apparatus within the nuclei of epithelial cells, and it appears to play a key role in the establishment and maintenance of epithelial phenotypes [72]. Joo *et al.* [73] studied the lncRNAs of the corneal epithelium by focusing on a small subset of lncRNAs that exhibit splicing changes in response to PNN knockdown. The results showed that the lncRNAs *SPACA6P*, *HAS2-AS1*, *RPARP-AS1*, *RP11-295G20.2*, and *NUTM2a-AS1* exhibited significant and reproducible expression changes and RNA processing after the perturbation of PNN expression. Although the findings are incomplete, they provide the first glimpse into the complexity and potential relevance of lncRNAs in the maintenance of epithelial cells, paving the way for further investigations into the roles of lncRNAs in cornea. In addition, Hoang *et al.* [74] performed an RNA-seq analysis and identified 86 differentially-expressed lncRNAs between lens epithelial cells and lens fiber cells; they included *RP23-237H8.2*, *AC135859.1*, *AL663030.1*, *AC128663.1*, and *AC100730.1*. Although the functional significance of these lncRNAs in lens development or physiology remains unknown, this comprehensive transcriptome analysis provides a valuable resource for the study of lens development, fiber differentiation, and lens pathogenesis [75].

## 3. Conclusions

To date, several lncRNAs have been implicated in eye development, including*Vax2os1*, *RNCR2*, *Six3OS*, *Tug1*, and *MALAT1*. These lncRNAs are recognized as important regulators of various processes, such as photoreceptor progenitor progression and retinal cell fate specification [14,59,76,77,78]. However, the roles of lncRNAs in the pathogenesis of ocular diseases are far from understood. Most of the lncRNAs mentioned in our review were identified by consulting relevant studies about diseases that share the same etiology or pathogenesis. For example, *BANCR* is involved in malignant melanoma and lung cancer; thus, researchers explored its role in retinoblastoma. *ANRIL* is significantly associated with increased susceptibility to type 2 diabetes [79]; thus, it is no surprise that this abnormally-expressed lncRNA may be relevant to the molecular mechanisms underlying diabetes complications. In addition, microarray analysis and RNA sequencing provide convenient but also comprehensive ways to identify aberrantly expressed lncRNAs. Undoubtedly, high-throughput RNA sequencing and computational analyses will substantially improve the characterization of noncoding RNAs to a much broader level than that of previous work.

As discussed above, many lncRNAs regulate specific facets of protein activity, thus, they may represent potential targets for drugs that are more refined and less toxic than conventional protein-targeting drugs. For instance, oligonucleotide antagonists specifically block the binding of oncogenic PRC2 to lncRNA, thereby inhibiting the repression of tumor suppressor genes. Oligonucleotides for the knockdown of deleterious lncRNAs have already been studied [21,80]. Although promising, this approach also has challenges, such as how to demonstrate efficient delivery accompanied by long-lasting effects on abnormal cells and how to evaluate toxicity, stability, and efficient targeting. Additional studies focusing on the druggability of known lncRNAs remain to be conducted.

Systematic identification of lncRNAs and a better understanding of their mechanisms of action can pave the way for early diagnosis and the design of better therapeutics. New candidate lncRNA genes and their molecular mechanisms remain to be explored. Focused studies will surely provide useful insights for understanding disease pathogenesis and identifying new disease mechanisms. Intensive research will inspire new hypotheses about pathogenesis and will lead to novel clinical applications.

## Figures and Tables

**Table 1:** Long noncoding RNAs (lncRNAs) involved in different types of ocular diseases.

Disease	lncRNA Name	Location	Function	Possible Mechanism	Reference
CN	*NR_033585*	Chr17	Pro-angiogenesis	NG	[24]
	*chr8:129102060–129109035 reverse strand*	Chr8	Anti-angiogenesis	NG	[24]
POAG	*ANRIL*	Chr9p21	Promotes retinal ganglion cell apoptosis	Act via TGF-β signal pathway or regulate neighboring genes CDKN2A/2B	[25,26,27]
PVR	*MALAT1*	Chr11q13	Promotes RPE proliferation, migration, and ERM formation	NG	[28]
DR	*MIAT*	Chr22q12	Promotes endothelial cell proliferation and migration	Act as ceRNA	[29]
	*MALAT1*	Chr11q13	Promotes endothelial cell proliferation, migration, and tube formation	Changes the levels of phosphorylated p38 MAPKs	[30,31,32]
CNV	*Vax2os1/os2*	Chr6	Highly expressed	Acts via RNA protein interaction	[33]
RB	*BANCR*	Chr9	Suppress proliferation, migration, invasion	Acts via the MAPK pathway/NF-κB pathway	[34]
	*MEG3*	Chr14q32	Inhibits proliferation induces apoptosis	Acts via the Wnt/β-catenin pathway	[35]
UM	*lncROR*	Chr18q21	Promote tumor growth and metastasis	Activates TESC promoter by epigenetic mechanisms	[36]
	*CRNDE*	Chr16q12	Promote cell growth and migration	Acts via the mTOR signalpathway/regulates gene expression by epigenetic mechanisms	[37]
MOMO syndrome	*LINC00237*	Chr20p11	Deficient in patient lymphoblasts	NG	[38]

CN: corneal neovascularization; POAG: primary open angle glaucoma; PVR: proliferative vitreoretinopathy; DR: diabetic retinopathy; CNV: choroidal neovascularization; RB: retinoblastoma; UM: uveal melanoma; NG: not given.
